# Dataset of search queries to map scientific publications to the UN sustainable development goals

**DOI:** 10.1016/j.dib.2021.106731

**Published:** 2021-01-09

**Authors:** Frederique Bordignon

**Affiliations:** Ecole des Ponts, Marne-la-Vallée, France

**Keywords:** Sustainable development goals, Bibliometrics, Search queries, Information retrieval

## Abstract

The dataset includes search queries that can be used to identify scientific publications related to the United Nations Sustainable Development Goals (SDGs). We propose a new approach to mitigate the polysemy of terms as much as possible by targeting the most relevant subject areas for each SDG. In addition, we also used a text-mining tool to identify as many relevant phrases as possible. Publications identified through this process cannot be considered as evidence of the commitment of authors and their institutions to actions towards the targets established by the UN. However, they can be an accurate indicator of which research is relevant to the issues addressed by the SDGs, whether or not it is a direct contribution.

## Specifications Table

**Table:** 

Subject	Library and Information Sciences
Specific subject area	Information retrieval, bibliometrics
Type of data	Search strings in txt files Tabular data in a csv file
How data were acquired	Search strings: manually created Survey data: collected with LimeSurvey
Data format	Raw, txt and csv files
Parameters for data collection	In order to mitigate the polysemy of terms as much as possible, we identified and targeted the most relevant subject areas for each SDG. In addition, we also used a text-mining tool (CorTexT) to identify as many relevant phrases as possible. The results were submitted (through LimeSurvey) to researchers for their feedback.
Description of data collection	These data were manually written in a text editor.
Data source location	Ecole des Ponts Marne-la-Vallée France
Data accessibility	Repository name: Mendeley Data Data identification number: http://dx.doi.org/10.17632/xrx7ddbbb4.1 Direct URL to data: http://dx.doi.org/10.17632/xrx7ddbbb4.1

## Value of the Data

• The annotated corpora resulting from these queries can be used for bibliometric analyses relying on the UN Sustainable Development Goals.
• Bibliometric experts can reuse these data to analyze existing corpora of scientific publications or to collect new corpora from bibliographic databases.
• These search queries can be used as calculated fields in Tableau Software but they can also be easily adapted to another tool by multiple search-and-replace actions.

## 1 Data Description

The United Nations define the Sustainable Development Goals (SDGs) as “;the blueprint to achieve a better and more sustainable future for all. They address the global challenges we face, including poverty, inequality, climate change, environmental degradation, peace and justice”; [1]. 17 SDGs were set in 2015 and intended to be achieved by the year 2030: Table 1

**Table 1:** UN Sustainable Development Goals (source: United Nations [1]).

(1) No Poverty	(10) Reducing Inequalities
(2) Zero Hunger	(11) Sustainable Cities and Communities
(3) Good Health and Well-being	(12) Responsible Consumption and Production
(4) Quality Education	(13) Climate Action
(5) Gender Equality	(14) Life Below Water
(6) Clean Water and Sanitation	(15) Life On Land
(7) Affordable and Clean Energy	(16) Peace, Justice, and Strong Institutions
(8) Decent Work and Economic Growth	(17) Partnerships for the Goals
(9) Industry, Innovation and Infrastructure	

In 2017, the UN defined specific targets and indicators for each goal [2]. Armitage et al. [3] argued that along with political action, research and development of technology are also essential for achieving these goals. While research for sustainable development is inherently directed at supporting sustainability-oriented societal change [4], it is valuable for research institutions to assess how they contribute to achieving the goals defined by the United Nations.

This is the reason why there have been several attempts to automatically identify scientific outputs related to these SDGs: they are based on rather complex Boolean queries combining keywords and phrases that are typical of the goals and targets defined by the United Nations policy text [3,[5], [6], [7], [8], [9]. These queries seem to us to be either too narrow because they stick to the UN text, or too broad because of the polysemy of some words, which led to false positive results. Some are too complex, too long, or with a proximity operator not available in tools such as Tableau Software (which can be helpful to bibliometrics experts when processing large amounts of data).

Nevertheless, they have been inspiring, especially those shared by Jayabalasingham et al. [10] and used by Elsevier in its tools and reports [6]. We propose a new approach to mitigate the polysemy of terms as much as possible by targeting the most relevant subject areas for each SDG. This allows to “;loosen”; the constraints of the queries without generating too many unwanted hits. In addition, we also used a text-mining tool to identify as many relevant phrases as possible. In other words, with this new method, we aimed at improving both precision and recall.

The dataset we share includes Boolean queries inserted in a conditional function (IF, THEN, ELSE). We have developed them with the aim of annotating structured corpora of scientific publications. They are therefore intended to be used directly in a tool such as Tableau Software. But as they are shared in txt format, they can be simply adapted for direct use in bibliographic databases such as the Web of Science, Scopus, Dimensions, or Lens.

The dataset contains one query per SDG. The function indicates that if the stated condition(s) is/are true then the SDG is assigned to the publication; otherwise the result is 0. Conditions are Boolean combinations of subject areas (ASJC codes) and terms.

Queries are proposed for SDGs 1 to 16. As [11] put it, the SDG “;Partnerships for the goals”; can be regarded as a meta-goal for implementing the SDGs. We consider that it could be assessed with an analysis of co-publications (e.g.: co-occurrence of countries mentioned in the authors' affiliations).

We would like to warn that publications identified through this process cannot be considered as evidence of the commitment of authors and their institutions to actions towards the targets established by the UN. However, they can be an accurate indicator of which research is relevant to the issues addressed by the SDGs, whether or not it is a direct contribution.

## 2 Experimental Design, Materials and Methods

### 2.1 Query design

**Fig. 1:**
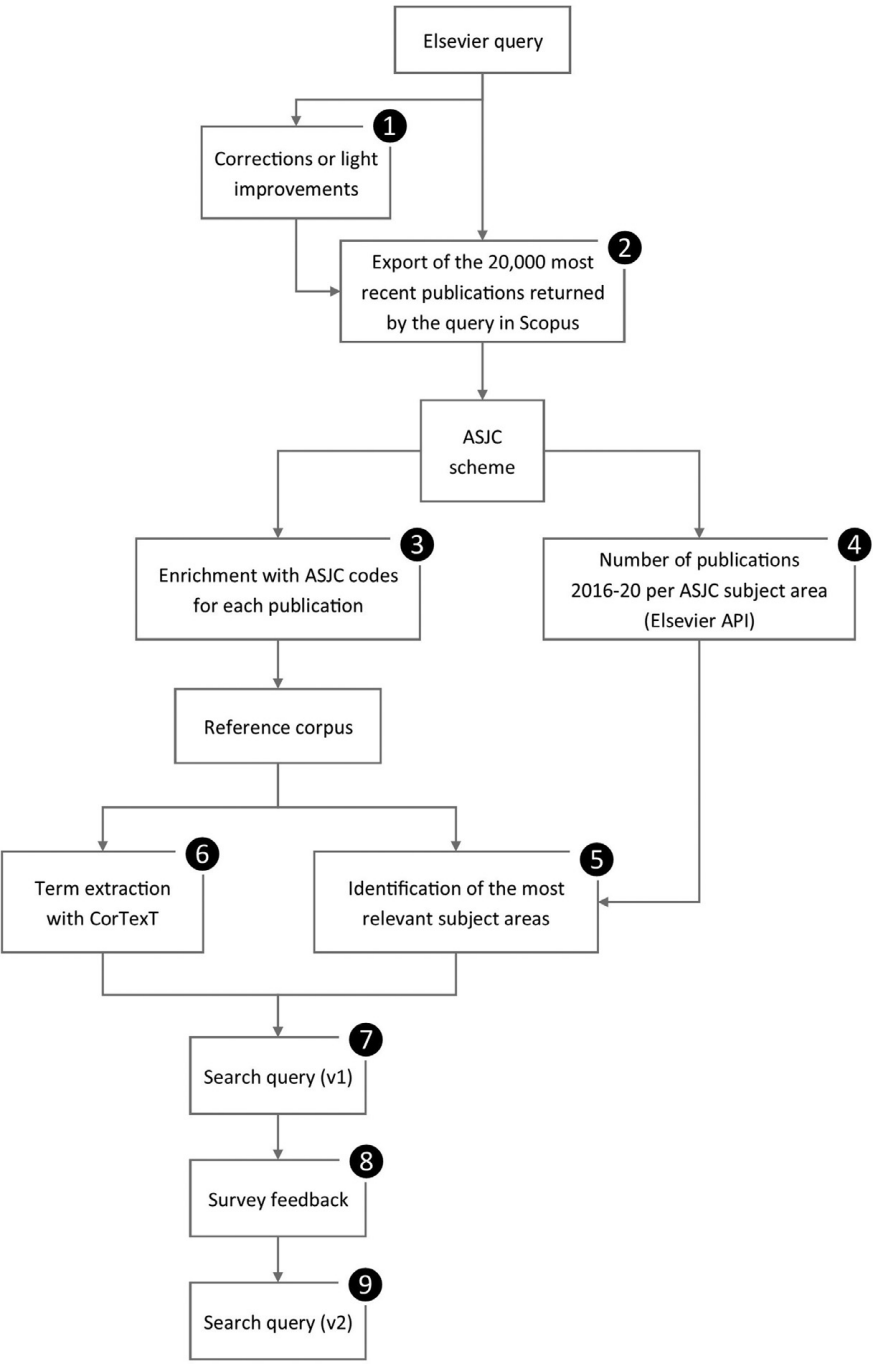
Workflow of the query design.

Each search string has been designed separately but according to the same basic principles presented in Fig. 1, the main steps of which are as follows:

1. We review and test the query proposed by the Elsevier team [10]. For a few SDGs, we make corrections (e.g. SDG 14) or improvements (e.g. SDG 7) before using the query with confidence.
2. With the query or a slightly updated version, we retrieve the 20,000 most recent publications from Scopus to build a reference corpus.
3. We use the All Science Journal Classification scheme (ASJC, see details below) to enrich each publication with the field(s) and subfield(s) assigned to the source it has been published in.
4. At the same time, thanks to Elsevier's API, we retrieve from Scopus the number of publications published between 2016 and 2020 in each ASJC subfield. Indeed, the number of documents varies a lot from one subfield to another [12] and makes it difficult to do comparisons.
5. To identify the most relevant subfields, we divide the number of publications in our reference corpus by the number of publications in Scopus (2016–2020). This normalization process allows us to determine in which subfields the publications are relatively more frequent and to consider whether they should be used in the query.
6. With CorTexT,^1^ a text-mining tool, we perform term extraction on Title, Abstract and Keywords of publications of the reference corpus.
7. We elaborate a new query combining ASJC subfield codes and extracted terms.
8. The positive results returned by the query (first version) are tested through a survey among researchers (see details below).
9. We propose an updated version of the search string, either confirming the first version or with improvements thanks to the survey participants' feedback.

While the survey we conducted helped to identify problems and to make an initial assessment of precision, it did not allow us to assess recall. In our survey, we found that some respondents were incorrect because they were unaware of all the targets related to a SDG. The title of the SDG and the brief description we provided could not be sufficient. Therefore, we think the recall can only be assessed by SDG experts.

If, depending on the tool, regular expressions cannot be used, attention should be paid to rather short words that can be found within others and thus generate unwanted hits. This is the case of *road*, which is found in many other words and in particular all those derived from *broad* for example, or *flake* in *snowflake* and *cornflake*, or *sea* in *disease, nausea* or *research*. To mitigate the problem and have simple queries, we use spaces and punctuation marks in our search strings.

In an effort not to overcomplicate the queries and to allow adjustments from other researchers, we have set aside lists of words and included them in variables. For example, some SDGs explicitly target developing countries by mentioning them in a very generic way:

(1) least developed countries, landlocked country, developing country, landlocked countries, developing countries, small island developing States, Africa

But these phrases are not satisfactory if the authors of a publication explicitly mention the name of the country. We, therefore, take advantage of a previous study [3] to retrieve a list of developing countries we include in a variable named [DevCountries]. This list has been constituted from the data made available by the UN on their website.

We have also created a variable named [Astronomy] with a list of words referring to planets in order to more easily exclude works in astronomy. This list can definitely be enriched.

Finally, in the same way, we have introduced a variable named [Infra] listing words referring to infrastructures, a list resulting from a text-mining operation we carried out in previous work [13].

### 2.2 All science journal classification (ASJC)

The All Science Journal Classification is a subject classification system elaborated by Elsevier to categorize journals (and also book series and conferences). It includes 27 fields and 334 subfields. They are all assigned a unique and permanent 4-digit code that we use in the queries proposed in this article. The nomenclature itself is stable with no new subject area since 2011. Nevertheless, the subjects assigned to the sources are regularly updated to stay in line with editorial changes in journals. The list of sources and subfield(s) assigned to them can be freely downloaded as an Excel file from Scopus database or Elsevier's website.^2^ The nomenclature with the description of the ASJC codes is available as a supplementary material.

### 2.3 Survey overview

As we stated earlier, the positive results returned by the first version of each query have been tested through a survey among researchers from Ecole des Ponts (*n* = 81, invited by mail) in order to confirm whether they are true or false positives. This online survey was used to test 99 sets of Title-Abstract-Keywords (randomly selected among publications from Ecole des Ponts researchers; also available in the dataset). For each set of title-abstract-keywords, the researcher was asked if the proposed SDG(s) (returned by the query under scrutiny) was/were relevant or irrelevant with the following question: “Do you think the following publication can be considered as a contribution to achieving the goals suggested below?”. The participant could also decide not to answer, and to choose “;I don't know”;.

Table 2 shows how many different answers had been gathered for each SDG and how many different respondents this corresponds to.

**Table 2:** Survey overview per SDG (answers, distinct respondents and participants who did not answer).

	Total number of answers			Percentage of answers			
SDG	Irrelevant	OK, relevant	All	Irrelevant	OK, relevant	Number of distinct respondents	Number of participants who did not answer
SDG1	11	46	57	19%	81%	39	9
SDG2	18	42	60	30%	70%	48	11
SDG3	33	80	113	29%	71%	54	10
SDG4	11	49	60	18%	82%	46	4
SDG5	6	47	53	11%	89%	38	6
SDG6	24	45	69	35%	65%	47	10
SDG7	82	78	160	51%	49%	52	19
SDG8	29	58	87	33%	67%	51	15
SDG9	13	34	47	28%	72%	35	6
SDG10	16	48	64	25%	75%	42	10
SDG11	37	147	184	20%	80%	61	24
SDG12	19	53	72	26%	74%	48	9
SDG13	25	47	72	35%	65%	36	13
SDG14	19	25	44	43%	57%	33	8
SDG15	25	21	46	54%	46%	35	6
SDG16	34	28	62	55%	45%	42	14

### 2.4 SDG 1: no poverty

We identify the most frequent subfields in the reference corpus; they are part of the 2 following fields:

- Social sciences (subfields: Development, Geography, Planning and Development, Public Administration, Demography, General Social Sciences, Sociology and Political Science, Political Science and International Relations)
- Economics, Econometrics and Finance.

They prove to be the most relevant for the topic, they are therefore fully included in the query and combined only with terms expressing poverty or referring to poor people (2)(2). We rule out the word *poor* on its own because it is an adjective that is also frequently used to describe the poor quality of something.

(1) poor population, the rural poor, urban poor area, rural poor area, poor children, poorer children, poorest children, poor women, poor and needy, poor borrower, poor workmanship, poor worker, poor working families…

These same words referring to the poor (2) are combined with a few specific phrases that could not be used on their own to return alone documents relevant for SDG 1 (e.g.: *food insecurity, income inequality* or *labor market*).

Finally, we enumerate expressions that explicitly refer to the fight against poverty. A large majority (81%) of respondents confirmed that the publications retrieved with this search query correctly reflect the topics of this SDG.

### 2.5 SDG 2: zero hunger

All publications from sources in *Agronomy and Crop Science* and *Food Science* seem to be relevant for this SDG. After several tests, we found that the subfield of *Soil science* is too broad, encompassing, for example, geotechnical research (which we think is more related to SDG 9), or urban runoff studies (which are more related to SDG 6). Then we combine polysemic expressions (e.g.: *food insecurity*) with the other subfields except medicine (which brings up too many publications about nutrition and dietetics), and finally we use a list of expressions that explicitly refer to the fight against hunger. 70% of the respondents confirmed the publications returned by this query are related to this SDG. Despite this consensus many respondents have erroneously considered works about climate extreme events as irrelevant. However, the United Nations do mention disasters as negative consequences on soil quality or food production.

### 2.6 SDG 3: good health and well-being for people

Before reusing the terms extracted by CorTexT, we modified Elsevier's initial query by removing *aids*, which was intended to retrieve works related to the fight against AIDS but which, in reality, also retrieved publications containing the verb *to aid* in the third person (3) or the noun *aid* in the plural (4).

(1) Also a microscopic examination of fungal culture aids in the identification.
(2) Representation of speech with high spectral resolution finds applications in the design of digital hearing aids and cochlear implants for people with hearing disability.

This problem resulted in too many unwanted publications in *Engineering*, as can be seen in Elsevier's report [6] in the graph dedicated to SDG 3. This being said, even after this correction and the use of CorTexT, it is difficult to list all the pathologies and cures that could be included in this SDG. We therefore stick to the Elsevier query, adding nevertheless *coronavirus* and *covid-19*. On the other hand, the query relies on subfields belonging to the fields of *Medicine* (*Medicine, Nursing, Dentistry, Health Professions, Immunology and Microbiology, Neuroscience, Pharmacology, Toxicology and Pharmaceutics*) or *Psychology*, and to the *Health* subfield of the *Social sciences* field. In addition, we dedicate a segment of the query to the medical system by intersecting the *Economics, Econometrics and Finance* field with some key expressions such as *hospitals* or *healthcare*.

A large majority (71%) of respondents confirmed that the publications retrieved by this query do reflect the theme of the SDG.

### 3.7 SDG 4: quality education

Subfields related to education exist in the ASJC scheme and they emerge as the most relevant thanks to our normalization operation: *Education, Developmental and Educational Psychology, Life-span and Life-course Studies, Cultural Studies*. Therefore, they are included in the query so that all publications from the sources belonging to them are associated with this SDG. The subfield of *Sociology and Political Science* also appears to be frequent, in order to better target the relevant publications, we simply combine it with the word *education*. Finally, CorText provides us with a list of unambiguous expressions to extend the query to other domains. 82% of respondents agreed that the proposed publications were related to this SDG.

### 3.8 SDG 5: gender equality

At first glance, the *Gender studies* subfield has the highest number of publications: 9% of the corpus of references in this SDG are related to it. But a closer look shows that including all publications from sources in this subfield would result in the inclusion of psychology studies that are out of the scope of this SDG. Therefore, the final search string is based on a list of phrases from Elsevier's query enriched by those found by CorTexT. 89% of respondents agreed that the proposed publications were related to this SDG.

### 3.9 SDG 6: clean water and sanitation

There is a great amount of papers about water-related issues, in many disciplines. Mining the text of the reference corpus has enabled the identification of unambiguous expressions:

(1) management of the water, water pollutant, polluted water, pollutants in water, pollutants from water, pollution of water, pollution in water, pollution water, pollution in the water, water and pollution, pollutants water, water by pollutants, water from pollutants, water purification, purification of water, water quality, quality of water, water recycling, recycling water, recycle water, water reuse

Other terms, such as monograms like *quality* or *pollution* are combined with *water* or *sanitation*. And of course all publications of sources in the subfield of *Water Science and Technology* are associated with SDG 6.

65% of respondents agreed that the proposed publications were related to this SDG but they did not perceive that publications dealing with groundwater pollution were indeed related to this SDG.

### 3.10 SDG 7: affordable and clean energy

The initial trials with Elsevier's query show that the phrase *solar energy* returns too many hits related to astronomy. They also show that it is too risky to use *renewable* alone which is an adjective that can be applied to many other things than energy:

(1) renewable neural implant, renewable material, renewable mandates

On the other hand, subfields related to medicine and life sciences have been excluded because the word *energy*, alone or in a phrase, can be polysemic:

(2) It is possible that a low energy diet could ameliorate the unfavorable effects of G allele of HSP70.
(3) Thus, the essential proteins associated with physiological activities were significantly upregulated, while those related to energy production were significantly downregulated.
(4) The unit energy consumption was predicted by artificial neural network (ANN), and the recovered moisture adsorption of activated alumina was measured by the dynamic adsorption test.

We disagree with [10] to include *life cycle assessment* in the search string for SDG 7. It seems to be more relevant for SDG 12 dedicated to responsible consumption and production. The main difficulty here lies in publications in chemistry, which we finally decided to include, since they encompass, for example, works on lithium-ion batteries, which are consistent with SDG 7 targets.

Despite these adjustments, only 49% of participants agreed to say publications submitted to them matched the SDG. Indeed, the bi-gram *energy conservation* leads to the retrieval of many false positives, mainly publications in mathematics mentioning a concept called *energy conservation* but irrelevant for SDG 7 targets. Therefore, rather than setting aside all publications in mathematics, we try to exclude those that mention both *energy conservation* and other terms that are strong signals that the topic is not related to energy storage:

(5) dissipative, dissipation, energy variable, energy–momentum conservation, pseudo-energy

### 3.11 SDG 8: decent work and economic growth

We first take all publications from *Organizational Behavior and Human Resource Management* and the 4 subfields included in economics (*General Economics, Econometrics and Finance, Economics, Econometrics and Finance (miscellaneous), Economics and Econometrics*) and combine them with words like *sustainable* or *sustainability*. We have chosen not to take all of the research in economics so as not to automatically include research about finance or marketing that is not related to sustainability nor is it related to decent work.

Another part of the query is constructed by intersecting the subfield of *Development* with work-related expressions such as:

(1) worker, workfare, employment, profession, employee, human resource, work participation, recruitment, workplace, career

The other part of the query is a list of unambiguous phrases directly related to SDG 8.

67% of respondents agreed that the publications retrieved with this query were related to this SDG.

### 3.12 SDG 9: industry, innovation, and infrastructure

For this query, we target works on infrastructure, innovation and industry using monograms in (12) combined with the *Development* subfield, which appears first in terms of percentage of publications in the reference corpus (after our standardization operation).

(1) innovation, industrial, industrialization, industrialization, industry, industries, manufacturing, infrastructure, enterprise, entrepreneur, [Infra]

We also target the publications from the following relevant subfields: *Industrial and Manufacturing Engineering, Management of Technology and Innovation* and *Computer Networks and Communications*. Furthermore, we take advantage of the following subfields to access the scientific outputs dedicated to infrastructure: *Civil and Structural Engineering, Building and Construction, Architecture, Transportation, Safety, Risk, Reliability and Quality, Geotechnical Engineering and Engineering Geology*. In order to limit unwanted hits, we combine them with terms referring to infrastructure ([Infra] variable).

Then, with CorTexT we find expressions that can enrich the original list from Elsevier. Finally, we remove the expression *traffic congestion* which overwrites all the results even though it is not central in the SDG.

72% of the participants confirmed that the publications returned by this query are relevant for this SDG.

### 3.13 SDG 10: reducing inequalities

This SDG is clearly cross-cutting, as indicated by the sentence that describes it in the UN's description: “;Reducing inequalities and ensuring no one is left behind are integral to achieving the Sustainable Development Goals.”; We therefore chose to keep close to the UN presentation text. Nevertheless, we pay particular attention to the words *migration, immigration, emigration*, and *migrant* which may have other meanings as examples in (13)(13) show:

(1) atom migration, migrant mesozooplankton, credit risk migration, migrant birds

To avoid out of the scope results, we cautiously combine those words with all subfields in *Economics, Econometrics and Finance* and the subfields of *Sociology and Political Science, Demography, Geography, Planning and Development, Political Science and International Relations*. The remaining part of the query is based on unambiguous expressions related to the SDG.

75% of the participants classified the publications we submitted to them as relevant.

### 3.14 SDG 11: sustainable cities and communities

We use the entire *Urban studies* subfield. For the subfields of *Waste Management and Disposal, Transportation* and *Geography, Planning and Development*, we combine them with words that restrict the topic of “;city”;:

(1) city, cities, human settlement, human settlements, urban, metropolitan, metropole, town, municipal, municipality, municipalities

We use these same words combined with others for the rest of the query which is based on a list of non-polysemic phrases.

A large majority (80%) of respondents confirms the query returns relevant publications for this SDG.

### 3.15 SDG 12: responsible consumption and production

We rapidly identify that publications from sources within the fields of *Environmental Chemistry, Environmental Engineering*, and *Waste management and disposal* must all be considered for this SDG. Then to avoid out-of-scope results, we target the field of *Environmental Science* for the part of the query based on *consumer behavior* and *behavioural economics*. Lastly, the remaining part of the query is a list of unambiguous expressions related to SDG 12.

74% of respondents agreed that the publications we submitted to them do relate to this SDG.

### 3.16 SDG 13: climate action

To begin with, all publications from sources related to the subfields of *Atmospheric science* and *Global and planetary change* are relevant to SDG 13. Then to target the issues of climate change within the publications from sources in the 13 subfields of the field of *Environmental science*, we apply to these subfields a weak constraint with the variants designating *climate* and *atmosphere*. Lastly, the query is completed by a list of unambiguous expressions on the topic. We considered it inappropriate to set aside the publications in *Paleontology* as the works in this field also contribute to research on current climate problems.

65% of respondents confirmed the publications we associated to this SDG are indeed relevant to its targets.

### 3.17 SDG 14: life below water

We identify the subfields of *Oceanography* and *Aquatic Science* as the most relevant and set up the search string to retrieve all publications related to them.

We take the position that *lakes, rivers, mangroves, deltas* etc. are also concerned even if the description of the SDG focuses on seas and oceans. But we are comforted in this choice by a UN publication on Lake Tanganyika highlighted on one of the SDG 14 web pages.

We also include in the query unambiguous *n*-grams, and more polysemic ones that we combine with the “;below water vocabulary”; 0 and exclude the fields of health and medicine.

(1) marine, submarine, maritime, ocean, sea, lake, delta, wetland, river, mangrove, estuary

57% of respondents agreed that the selected publications were related to this SDG. Some of the publications they rejected were about the study of waves. We admit that we do not have any certainty on this issue, in any case it must depend on the related topics dealt with in these papers.

### 3.18 SDG 15: life on land

First we rule out the specific subfield of *Astronomy and Astrophysics*. Then, we target publications from sources associated with the following subfields: *Nature and Landscape Conservation, Forestry, Earth-Surface Processes, Soil Science, Animal Science and Zoology, Insect Science* but we exclude publications mentioning the “;below water vocabulary”; of SDG 14 (15).

Then we develop a list of unambiguous expressions that we look for in all subfields except medicine and health. Some phrases could also apply to SDG 14, thus, in order to avoid unwanted results, we combine them with words expressing life on land.

54% of the participants reported that the publications we had suggested for this SDG did not match. This allowed us to identify a very difficult problem to deal with regarding the words *forest* or *forestry*. They have a very common meaning in computer science. As a consequence, we have improved the query by adding a list of words to be excluded:

(1) tree set, tree set, magnetic resonance, wireless sensor, tree graph, stack tree, decision tree, poset, operad, algebra, tree-wise, tree wise, tree of shapes, mathematics, tree structured data, tree-structured data

### 3.19 SDG 16: peace, justice and strong institutions

For this query, we select all publications related to the following subfields that have been identified as the most relevant*: Health (social science), Law, Safety Research, Sociology and Political Science, Political Science and International Relations, Public Administration*.

In the second part of the query, we enumerate a range of specific expressions and combine them with all the fields that are not related to medicine.

This SDG was very problematic as 55% of the respondents rejected our proposals. These rejections are mainly due to the inclusion of the *Sociology and Political Science* subfield. More data should to be collected to make a more refined assessment in order to measure the benefits and drawbacks of including this field. We can suggest to adjust the query according to the corpus of publications being reviewed.

## Ethics Statement

The recruitment of participants was carried out via the internal mail servers of Ecole des Ponts without any specific targeting other than the researchers of the institution. Moreover, access to the questionnaire was via a unique independent URL (i.e.: no token was used and therefore there is no possibility to link the participant to his/her set of answers in LimeSurvey). Respondents’ involvement was completely anonymous and voluntary; they had to give their informed consent for inclusion before they could participate in the survey. They were informed that the survey was anonymous and that no personal data was collected. Only the answers to the questions were collected.

## Declaration of Competing Interest

The author declares that she has no known competing financial interests or personal relationships, which have, or could be perceived to have, influenced the work reported in this article.

## Appendix Supplementary materials

Image, application 1

## Footnotes

2 https://www.elsevier.com/solutions/scopus/how-scopus-works/content

## Footnotes

1 https://www.cortext.net
